# Effect of targeted ovarian cancer immunotherapy using ovarian cancer stem cell vaccine

**DOI:** 10.1186/s13048-015-0196-5

**Published:** 2015-10-24

**Authors:** Di Wu, Jing Wang, Yunlang Cai, Mulan Ren, Yuxia Zhang, Fangfang Shi, Fengshu Zhao, Xiangfeng He, Meng Pan, Chunguang Yan, Jun Dou

**Affiliations:** Department of Pathogenic Biology and Immunology, School of Medicine, Southeast University, Nanjing, 210009 China; Department of Gynecology & Obstetrics, Zhongda Hospital, School of Medicine, Southeast University, Nanjing, 210009 China; Department of oncology, Zhongda Hospital, Southeast University, Nanjing, 210009 China; Department of Medical Oncology, Affiliated Tumor Hospital of Nantong University, Nantong, 226361 China

**Keywords:** Epithelial ovarian cancer, Cancer stem cells, Vaccine, Antitumor immunity

## Abstract

**Background:**

Accumulating evidence has shown that different immunotherapies for ovarian cancer might overcome barriers to resistance to standard chemotherapy. The vaccine immunotherapy may be a useful one addition to conditional chemotherapy regimens. The present study investigated the use of vaccine of ovarian cancer stem cells (CSCs) to inhibit ovarian cancer growth.

**Methods:**

CD117^+^CD44^+^CSCs were isolated from human epithelial ovarian cancer (EOC) SKOV3 cell line by using a magnetic-activated cell sorting system. Pre-inactivated CD117^+^CD44^+^CSC vaccine was vacccinated into athymic nude mice three times, and then the mice were challenged subcutaneously with SKOV3 cells. The anti-tumor efficacy of CSC vaccine was envaluated by in vivo tumorigenicity, immune efficient analysis by flow cytometer, and enzyme-linked immunosorbent assays, respectively.

**Results:**

The CD117^+^ CD44^+^CSC vaccine increased anti-ovarian cancer efficacy in that it depressed ovarian cancer growth in the athymic nude mice. Vaccination resulted in enhanced serum IFN-γ, decreased TGF-β levels, and increased cytotoxic activity of natural killer cells in the CD117^+^ CD44^+^CSC vaccine immunized mice. Moreover, the CSC-based vaccine significantly reduced the CD117^+^CD44^+^CSC as well as the aldehyde dehydrogenase 1 positive cell populations in the ovarian cancer tissues in the xenograft mice.

**Conclusion:**

The present study provided the first evidence that human SKOV3 CD117^+^ CD44^+^CSC-based vaccine may induce the anti-ovarian cancer immunity against tumor growth by reducing the CD117^+^CD44^+^CSC population.

## Background

Epithelial ovarian cancer (EOC) is the leading cause of death from gynecologic malignancy in the China. Most asymptomatic early stage patients are lack of early diagnostic tools, thus the disease is usually diagnosed in a late stage. Despite ovarian cancer a highly chemosensitive disease, it is only infrequently cured. One of the main reasons lies in the presence of drug-resistant cancer stem cells (CSCs) that represent a subset of cells in the bulk of tumors and play a key role in the onset of tumor recurrence, distant metastasis, and drug-resistance [1, 2]. In EOC, CD117^+^CD44^+^cell phenotypes express CSC markers, and can survive conventional therapies such as chemotherapy, and give rise to recurrent tumors that are more chemo-resistant and more aggressive [2, 3]. Thus, novel approaches to CSC therapy are needed urgently to address this clinical need.

Accumulating evidence has suggested that the immune system has its ability to recognize and eliminate microscopic disease, and it may be paramount in preventing tumor recurrence. Ovarian cancer vaccines that target tumors through inducing immune responses against tumor cells, are a promising novel immunotherapy strategy addition to the treatment of ovarian cancer. However, ovarian cancer-specific vaccines have demonstrated minimal clinical efficacy in patients with established drug-resistant and metastasis disease [4, 5]. Emerging study suggests that the addition of immunotherapy to existing therapeutic options could lead to a great improvement in the outcome of ovarian cancer immune tolerance, especially when targeting CSCs [6]. Thus, vaccination directed at CSCs may broaden the antigenic breadth and function as a tumor-associated antigen, and stimulate the immune responses against autologous ovarian cancer cells [7, 8]. Towards this end, we used the previously identified EOC CSCs that have the CD117^+^CD44^+^cell phenotypes in human EOC SKOV3 cell line [2, 3, 9, 10] to investigate the therapeutic potential of this vaccine for targeting EOC CSCs in the study.

Here we showed that the SKOV3 CD117^+^CD44^+^CSC vaccine elicited strongly anti-ovarian cancer immune responses that significantly led to suppressing tumor growth, decreasing CD117^+^CD44^+^CSC and aldehyde dehydrogenase 1 (ALDH1) positive cell populations in tumor tissues in the vaccinated nude mice. This CSC vaccine provided a potential anti-ovarian cancer regimen for inhibiting EOC CSC’s growth in mice.

## Materials and methods

### Cell lines and mice

Human EOC SKOV3 cell line was acquired from an ovarian cancer patient, which is a well-established ovarian cancer model system; YAC-1 cell line is Moloney leukemia-induced T-cell lymphoma of A/Sn mouse origin. These cell lines were purchased from the Cellular Institute in Shanghai, China. Cells were cultured in complete media consisting of RPMI 1640, 2 mM L-glutamine, 100 U/ml penicillin, 100 μg/ml streptomycin, and 10 % fetal bovine serum (FBS). The medium was refreshed every 3 days to maintain adherent cells. When SKOV3 cells reached 90 % confluence, cells were harvested with 0.25 % trypsin-1 mM EDTA (Sigma- Aldrich, St. Louis, MO, USA) treatment for 2 mins. YAC-1 cells were conditional cultured and passaged in RPMI 1640 medium.

Balb/c athymic nude mice of 5–6 weeks of age were acquired from the Animal Center of Yang Zhou University of China (license number: SCXK, Jiangsu province of China, 2007–0001) and were raised under sterile conditions in air-filtered containers at the Experimental Animal Center, School of Medicine, Southeast University. All the experiments were performed in compliance with the guidelines of the Animal Research Ethics Board of Southeast University, China. Full details of approval of the study can be found in the approval ID: 20080925.

### Isolation of CD44^+^CD117^+^cells

CD44^+^CD117^+^cells were isolated from the SKOV-3 cell line using the magnetic-activated cell sorting (MACS) method that was performed as described previously [10, 11]. Briefly, CD44^+^subsets were first isolated using the mouse antihuman CD44 antibody coupled to magnetic microbeads (code number: 130-095-194, antibody dilution, 1:20, Miltenyi Biotec., Bergisch Gladbach, Germany) and followed by the magnetic column selection or depletion. The resulting cells were then depleted of CD117 negative subsets using mouse antihuman CD117 antibody coupled to magnetic microbeads (code number: 130-091-332, antibody dilution, 1:20, Miltenyi Biotec., Bergisch Gladbach, Germany). The CD44^+^CD117^+^cells were named for the EOC cancer stem cells as ‘EOC SKOV-3 CD44^+^CD117^+^CSCs’, and the resulting cells were named for the EOC non-cancer stem cells as ‘EOC SKOV-3 non-CD44^+^ CD117^+^ CSCs’ [3, 10–12]. The isolated cells were placed in stem cell culture medium by resuspension in serum-free DMEM/F12 supplemented with 20 ng/mL human recombinant epidermal growth factor (Invitrogen, CA, USA), 10 ng/mL basic fibroblast growth factor (Invitrogen, CA, USA), 5 μg/mL insulin (Sigma-Aldrich, Missouri, USA), and 0.5 % bovine serum albumin (Sigma- Aldrich, Missouri, USA) [13, 14]. The isolated CD44^+^CD117^+^CSCs were further identified by using a flow cytometer (FCM, BD, USA) [15].

### Mouse immunization protocol

Balb/c nude mice were used to assess the in vivo CSC vaccine efficacy. Twelve mice (female, weight: 16–18 g and age between 5 and 6 weeks) were randomly divided into four groups of equal size (three per group): the SKOV3 CD117^+^CD44^+^CSC group, the SKOV3 non-CD117^+^ CD44^+^CSC group, the SKOV3 cell group, and the phosphate-buffered saline (PBS) group. The nude mice received subcutaneous vaccination in the right flank with mitomycin C (50 μg/ml) inactivated above different vaccines (5 × 10^4^) three times, an interval of 14 days between the immunizations. All immunized mice were challenged subcutaneously with 5 × 10^6^ SKOV3 cells 10 days after final vaccination. Tumor formations in each mouse was monitored every 3-5 days by taking 2-dimensional measurements of individual tumors, and then the tumor-free mice were observed, respectively [16]. Mice were also monitored for the general health indicators such as overall behavior, feeding, body weight and appearance of fur after vaccination. The endpoint for this study was one diameter of tumor ≥20 mm, at which point mice were euthanized. Vaccine immunization and in vivo tumorigenicity experiment was repeated twice.

### Enzyme-linked immunosorbent assay (ELISA)

Fresh blood from all mouse groups was obtained before sacrificing by anesthesia. Serum levels of interferon-γ (IFN-γ) and transforming growth factor-β (TGF-β) was measured using a commercially available ELISA kits according to the manufacturer’s protocol (eBioscience, San Jose, CA, USA). Briefly, the serum samples were diluted at 1:10, and each cytokine was captured by the specific primary antibody and detected by biotin-labeled secondary antibody. Plate was read at 450/570 nm using a microplate reader (Bio-Rad Labs, Hercules, CA, USA). Samples and standards were run in triplicate, and the sensitivity of the assay was 0.1 units/ml for IFN-γ and TGF-β. The Kit is suitable for detecting samples that include cell culture supernatant and serum [17, 18].

### NK cytotoxicity

At the end of the experiments, the spleen tissues were harvested from the immunized mice. 5 × 10^6^ splenocytes were labeled with 0.5 mM 5-(and 6)-carboxy-fluorescein diacetate succinimidyl ester (CFSE; 20 μg/ml) at 37 °C for 20 mins. Splenocytes were washed twice in PBS containing 5 % FBS to sequester any free CFSE. The CFSE-labeled splenocytes as effector cells were seeded with a constant number of YAC-1 target cells in a 96-well plate at 25:1 ratios of effector cells to target cells. Flow cytometric CFSE/7-AAD cytotoxicity assay was analyzed by FCM [19, 20].

### Quantitative real-time reverse transcription-PCR (qRT-PCR)

qRT-PCR analysis was performed on an ABI step one plus real-time system (Applied Biosystems). Total cellular RNA was isolated from each sample by using a Qiagen RNeasy Kit (Qiagen, Valencia, CA). One microgram of total RNA from each sample was subjected to cDNA synthesis using the Superscript III reverse transcriptase (Invitrogen). cDNAs were amplified by PCR with primers as follows: Perforin (sense, 5′-TCCTATGGCACGCACTT TATCAC-3′; antisense, 5′-TCCACGTTCAGGCAGTCTCCTAC-3′); Granzyme B (sense, 5′-GCTGCTAAAGCTGAAGAGTAAGG-3′; antisense, 5′-GCGTGTTTGAGTATTTGCCC A TT-3′); TGF-β (sense, 5'-TGGAAACCCACAACGAAATCT-3′; antisense, 5'-GCTGAGGT ATCGCCAGGAAT-3′); β-actin (sense, 5′-TTTCCAGCCTTCCTT CTTGGGTAT-3′; antisense, 5′-TGTTGG CATAGAGGTCTTTACGG-3′). The mRNA levels of the genes of interest were expressed as the ratio of each gene of interest to β-actin for each sample. SYBR Green quantitative PCR amplifications was performed in the Step one plus Detection System (Applied Biosystems). The comparative Ct (ΔΔCt) method was used to determine the expression fold change [3].

### Analysis of CD44^+^CD117^+^CSC population in tumor tissues

The ovarian cancer tissues were harvested from the mice immunized with the different vaccines at the end of the experiments, and were developed into cell suspension that were used to analyze the CD44^+^CD117^+^CSC population by FCM assay. Briefly, a total of 2 × 10^5^ tumor cells were suspended in PBS and labeled with anti-Human/Mouse CD44 fluorescein isothiocyanate (FITC) 1:100 (eBioscience, CA, USA), and anti-Human CD117 phycoerythrin (PE) 1:20 (eBioscience, CA, USA) antibodies for immunofluorescence detection. Equal number of the cells cultured in stem cell culture medium was analyzed by FCM with Beckman Coulter Cell Quest software [9, 21].

### Analysis of ALDH1 activity in cells

Analysis of ALDH1 activity in cells was performed using a commercially ALDEFLUOR kit (StemCell Technologies, Durham, NC, USA) according to the manufacturer’s protocol as described in the published papers [1, 22]. Briefly, cells obtained from freshly dissociated ovarian cancer tissues from the mice immunized with the different vaccines were suspended in ALDEFLUOR assay buffer containing ALDH substrate (BAAA, 1 μmol/l per 1 × 10^6^ cells) and incubated during 45 mins at 37 °C. As negative control, each sample of cells an aliquot was treated with 50 mmol/l diethylaminobenzaldehyde (DEAB), a specific ALDH inhibitor. To clear cells of mouse origin from the xenotransplanted tumors, we used staining with an anti-H2Kd antibody (BD biosciences, 1/200, 30 min on ice) followed by staining with a secondary antibody labeled with PE (Jackson labs, 1/250, 30 min on ice). The sorting gates were established using as negative controls. For viability, the ALDEFLUOR-stained cells treated with DEAB and the staining with secondary antibody alone. Analysis was performed by using a FCM (BD, USA) [9, 19].

### Statistical analysis

Values of interest were presented as the average of ± S.D. for at least three independent experiments. Differences between the test and the control conditions were assessed by Student’s *t* test analysis. Bonferroni correction was used where multiple comparisons were made. Statistically significant difference is indicated by: * when *p* < 0.05, ** when *p* < 0.01 and *** when *p* < 0.003.

## Results

### SKOV3 CD117^+^CD44^+^CSC vaccine inhibits the ovarian cancer growth in the vaccinated nude mice

**Fig. 1:**
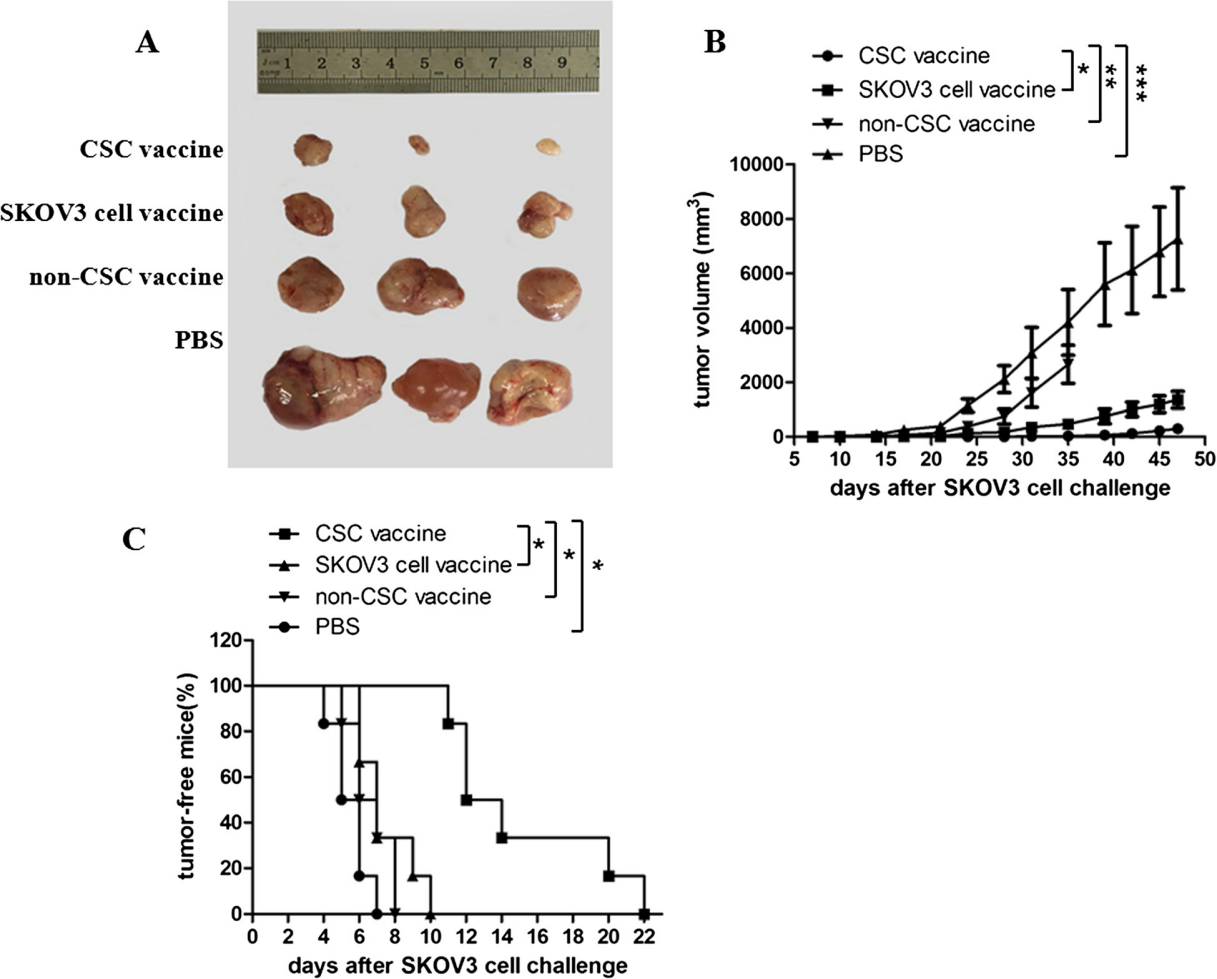
CD117^+^CD44^+^CSC vaccine exhibits a potent antitumor activity in mouse ovarian cancer model. **a** Images exhibits the tumor sizes dissected from the vaccineated mice 47 days after mice were challenged with SKOV3 cells. The nude mice received subcutaneous vaccination in the right flank with 50 μg/ml mitomycin C inactivated 5 × 10^5^ different cell vaccines respectively. A vaccination cycle is consisted of three vaccinations at a biweekly interval. Alive SKOV3 cells (5 × 10^5^) were injected subcutaneously in the left flank 10 days after the final vaccination; **b** The dynamic state changes of tumor volumes in SKOV3 ovarian cancer beraing nude mice; **c** Tumor free nude mice challenged with SKOV3 cells

In this study, we first wanted to know whether the SKOV3 CD117^+^CD44^+^CSC vaccine would elicit an immune response against SKOV3 ovarian cancer in nude mouse model. Figure 1a shows that images of tumor sizes on day 47 after the immunized mice were challenged with SKOV3 cells. It was found that the all mice immunized with the CD117^+^CD44^+^CSC vaccine grew tumors in 22 days but the tumor volume was statistically significant decreased compared with the mice immunized with the SKOV3 cell vaccine (*p < 0.05) or the non-CD117^+^ CD44^+^CSC vaccine (**p < 0.01). The time of tumor occurrence in the CD117^+^CD44^+^CSC vaccined mice was also markedly postponed in contrast to the mice immunized with the non-CD117^+^CD44^+^CSC and the SKOV3 cell vaccines (*p < 0.05), which are shown in Fig. 1b and c. Whereas the tumor sizes from the mice immunized with PBS (Fig. 1a) were bigger than that of mice immunized with the CD117^+^CD44^+^CSC (***p < 0.003); the time of tumor occurrence was also earlyer than that of mice immunized with the other vaccines (*p < 0.05). From these results, we concluded that the SKOV3 ovarian cancer growth was significantly inhibited in the mice vaccinated with the SKOV3 CD117^+^CD44^+^CSC-based vaccine.

### SKOV3 CD117^+^CD44^+^CSC vaccine elicits a strong immune responses in vaccinated nude mice

**Fig. 2:**
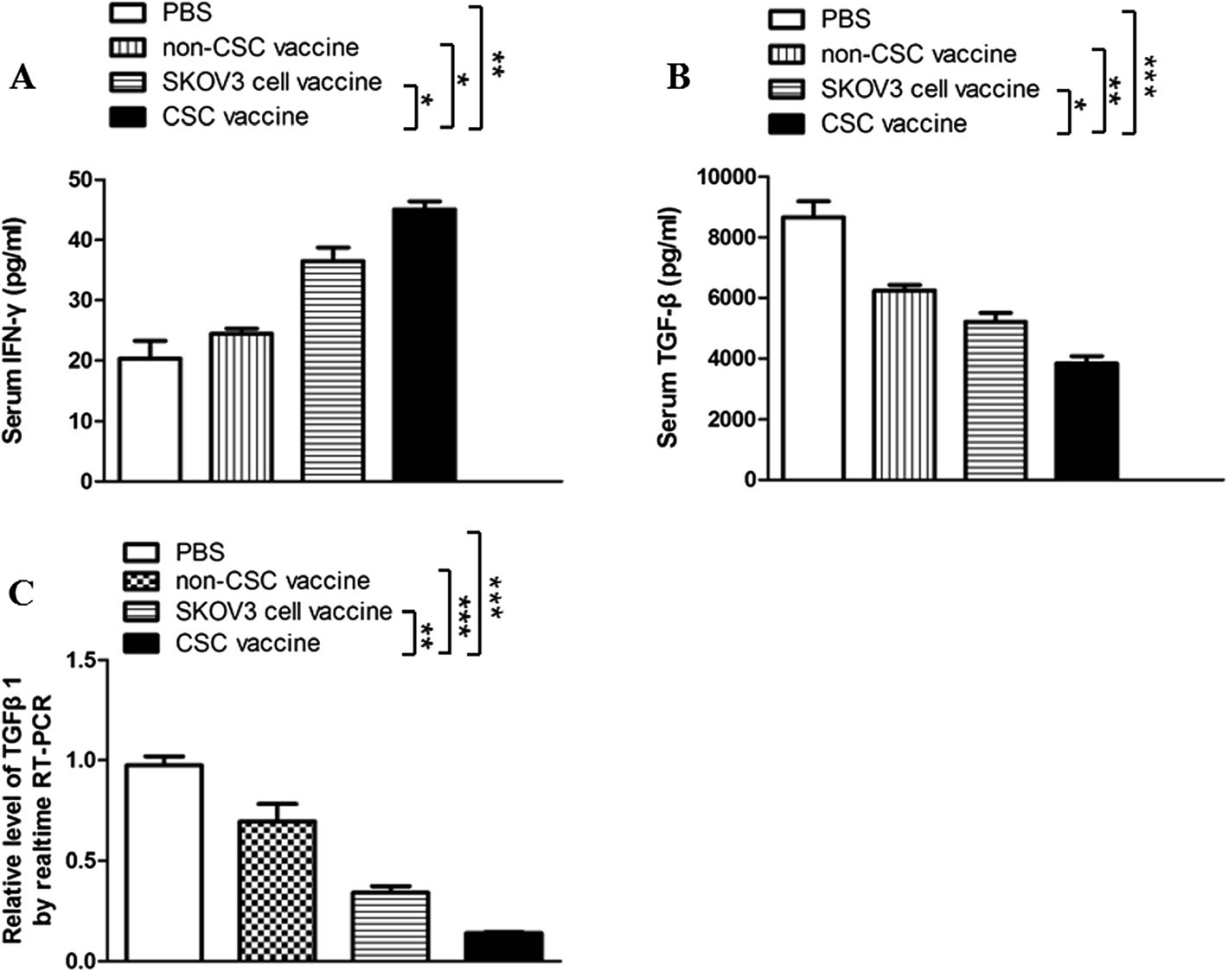
Levels of IFN-γ and TGF-β in the vaccinated mice challenged with SKOV3 cells. Serum levels of IFN-γ and TGF-β were tested by enzyme linked immunosorbent assay. The mice were immunized subcutaneously with the inactivated different vaccines and then were challenged by the SKOV3 cells as described in the section of materials and methods. **a** Serum IFN-γ level in a various vaccine groups; **b** Serum TGF-β level in a various vaccine groups; **c**. Ovarian cancer tissue TGF-β level in a various vaccine groups. *p <0.05, **p <0.01 and ***p <0.003; refer to the statistically significant differences as indicated. Data are represented as mean +/− SEM (*n* = 6)

To evaluate the immune efficacy of the SKOV3 CD117^+^CD44^+^CSC-based vaccine, we tested the levels of IFN-γ and TGF-β. Figure 2a shows the serum IFN-γ level was significantly increased in the CD117^+^CD44^+^CSC vaccine group compared with the non-CD117^+^CD44^+^CSC vaccine group (*p < 0.05) or SKOV3 cell vaccine group (*p < 0.05) or PBS group (**p < 0.01). Whereas the TGF-β level was significantly decreased in the CD117^+^CD44^+^CSC vaccine group in contrast to the SKOV3 cell vaccine (*p < 0.05) and non-CD117^+^CD44^+^CSC groups (**p < 0.01), and PBS group (***p < 0.003), respectively, as is shown in Fig. 2b. Similarly, the tumor tissue TGF-β level was markedly reduced in the CD117^+^CD44^+^CSC vaccine group in contrast to the control groups as is shown in Fig. 2c. These consistent cytokine level changes may be a helpful for eliciting the immune responses against SKOV3 ovarian cancer in nude mice.

### Comparison of the cytotoxicity of NK cells between the different vaccines immunized mice

**Fig. 3:**
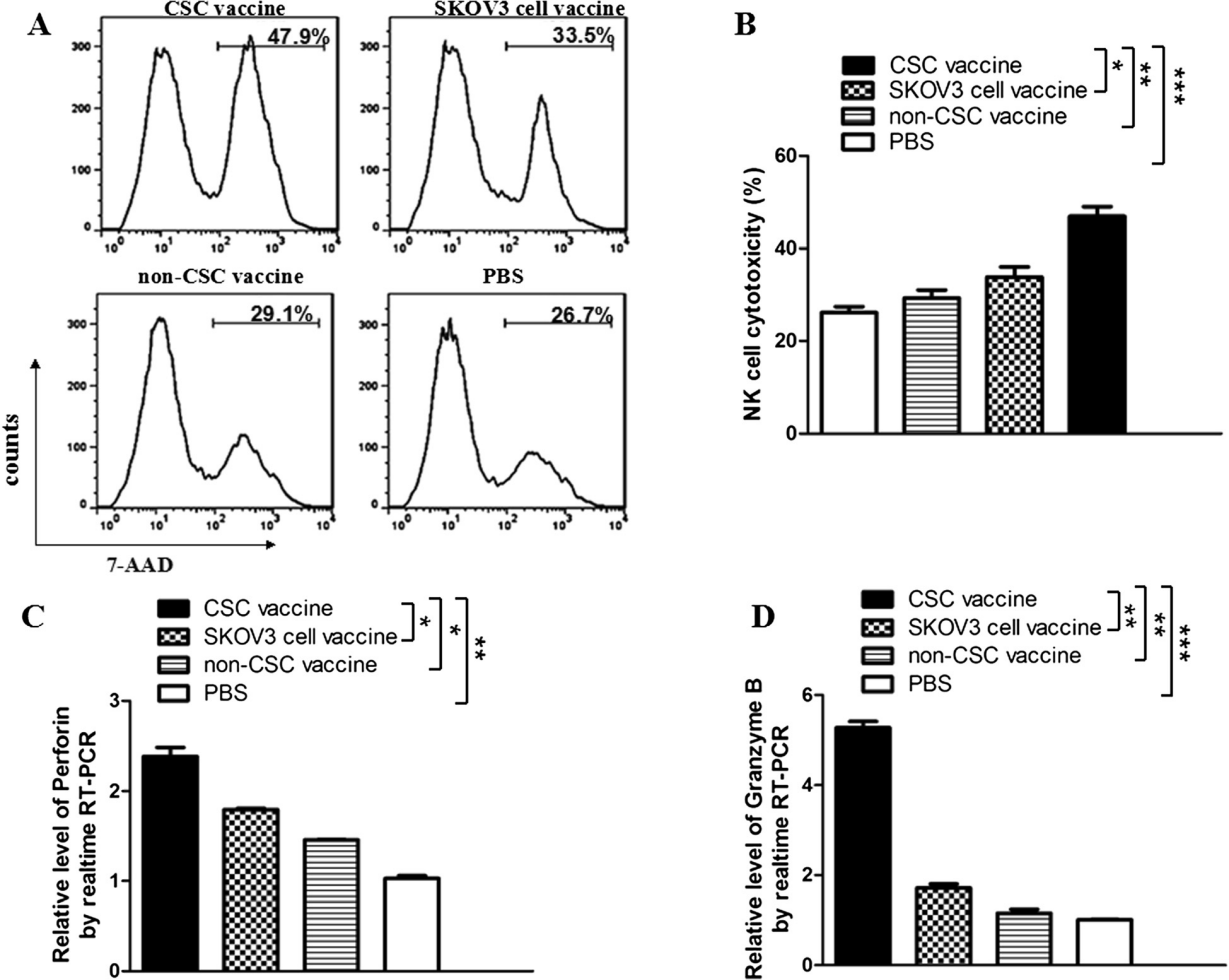
Vaccination with CD117^+^CD44^+^CSC vaccine induced NK cell response against SKOV3 ovarian cancer. **a** FCM analysis results indicate the cytotoxicity of NK cells in mice immunized with the different vaccines, respectively. The ratio of the effector cells (splenocytes from the immunized mice) to the target cells (YAC-1cells) was 25:1; **b** A representative set of data for six mice were used to detect NK cytotoxicity; **c** and **d** The analysis results of qRT-PCR show the expression of perforin and granzyme B in ovarian cancer tissues, respectively. An experiment was repeated twice. Statistically significant differences are indicated by asterisk for *p < 0.05, **p < 0.01, and ***p < 0.003, respectively

Since the cytotoxic activity of immune cells following administration of xenogeneic cancer vaccine in mice may represent an antitumor immunity efficacy, we therefore analyzed the NK cytotoxicity in the vaccinated mice in four separated experiments. Figure 3a gives the NK cytotoxicity (splenocytes against target cells YAC-1) in the SKOV3 CD117^+^CD44^+^CSC vaccine group was the highest (49.9 %) among 4 group vaccines, and the SKOV3 non-CD117^+^ CD44^+^CSC vaccine group ranked second (33.5 %). The NK cytotoxic activity was the lowest in the PBS group (26.7 %). There were a significant differences between the SKOV3 CD117^+^ CD44^+^CSC and SKOV3 vaccine groups (*p < 0.05), between the SKOV3 CD117^+^CD44^+^CSC and the SKOV3 non-CD117^+^ CD44^+^CSC vaccine groups (**p < 0.01), and between the SKOV3 CD117^+^CD44^+^CSC vaccine and PBS groups (**p < 0.01) as is shown in Fig. 3b.

NK cell performance of the cytotoxicity against target cells mainly depends on releasing effective molecules, perforin and granzyme B, so we further tested the expression of the two molecules in ovarian cancer tissues. Consistently, the expression of perforin and granzyme was significantly increasd in the SKOV3 CD117^+^CD44^+^CSC vaccine group compared with other control groups, which was statistically significant (Fig. 3c and d).

### Analysis of the CD44^+^CD117^+^cell as well as ALDH-positive cell populations in vaccinated mice challenged with SKOV3 cells

**Fig. 4:**
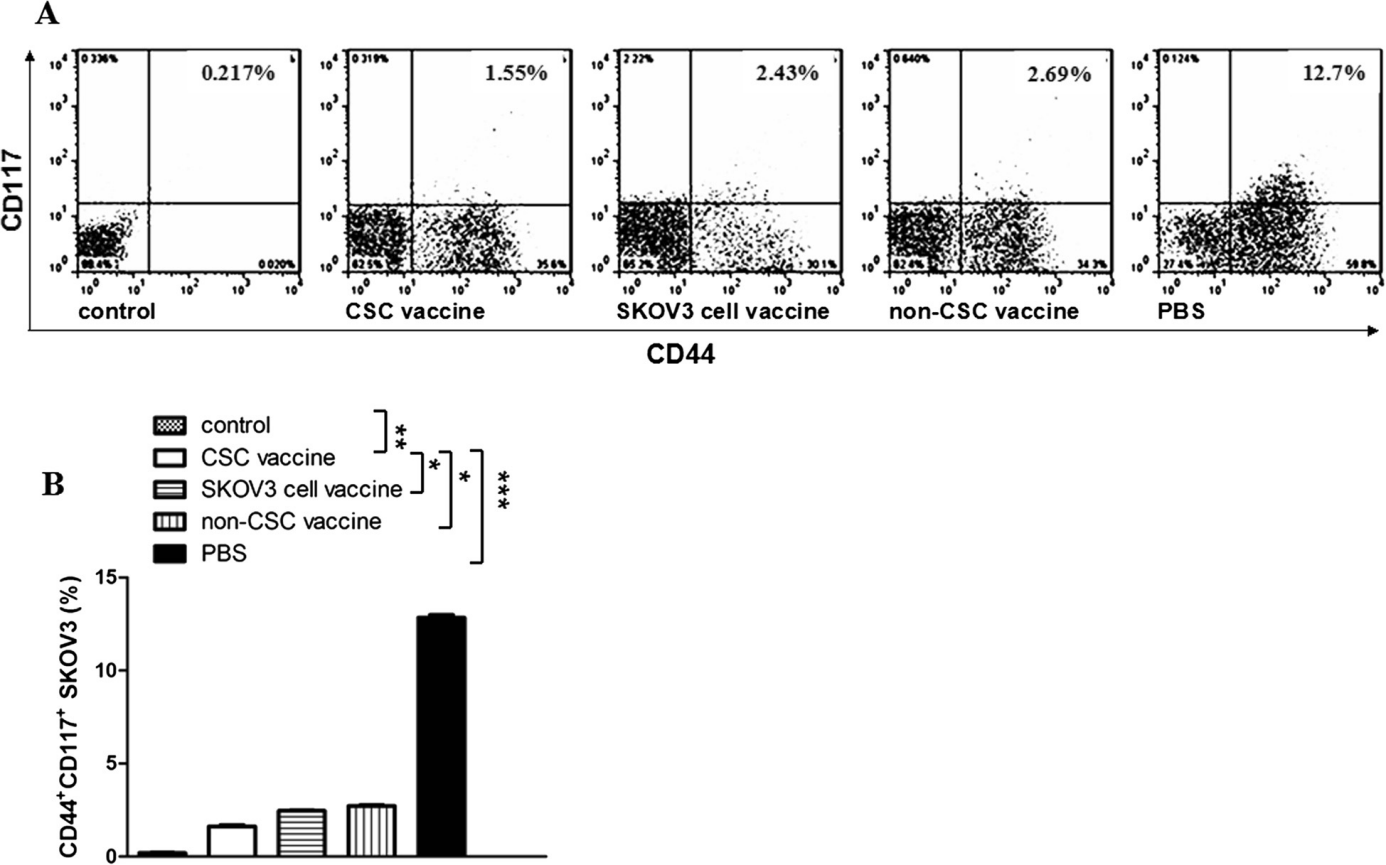
CD44^+^CD117^+^cell population in ovarian cancer tissues in nude mice. 2 × 10^5^ ovarian cancer tissue cell suspension in PBS were labeled with anti-Human/Mouse CD44 FITC 1:100, and anti-Human CD117 PE 1:20 antibodies for immunofluorescence detection. **a** FCM analysis shows that the number of CD44^+^CD117^+^double positive cells in CD117^+^CD44^+^CSC vaccine group was the lowest among 4 groups, whereas this number was the highest in PBS group; **b** Quantitative analysis of CD44^+^CD117^+^double positive cells in ovarian cancer tissues from the mice immunized with the different vaccines. *p <0.05, **p <0.01 and ***p <0.003; refer to the statistically significant differences as indicated

To assess the targeted effect of the CSC vaccine on elimination of CD44^+^CD117^+^CSCs, we investigated the CD44^+^CD117^+^double positive cell population in ovarian cancer tissues from the vaccinated mice. Figure 4a gives that in the PBS immunized mice, CD44^+^CD117^+^double positive cells were accounting for 12.7 % that was the highest among 4 groups, which was statistically significant compared with the SKOV3 CD44^+^CD117^+^CSC vaccine group (1.55 %, ***p <0.003); whereas the CD44^+^CD117^+^CSC vaccine group and the non-CD44^+^ CD117^+^CSC vaccine group had different CD44^+^CD117^+^cell population rates(1.55 % in the CSC vaccine group vs 2.43 % in the SKOV3 cell vaccine group; see Fig. 4a). The difference was statistically significant (*p < 0.05, Fig. 4b). In our previous studies, the CD44^+^CD117^+^double positive cell population in the SKOV3 cell line was only around 3.1–4.2 % [9, 11], and we think that the CD44^+^CD117^+^double positive cell population in ovarian cancer tiuuses from the CD44^+^CD117^+^ CSC immunized mice was significantly decreased compared with the mice immunized with other SKOV3 cell vaccine .

**Fig. 5:**
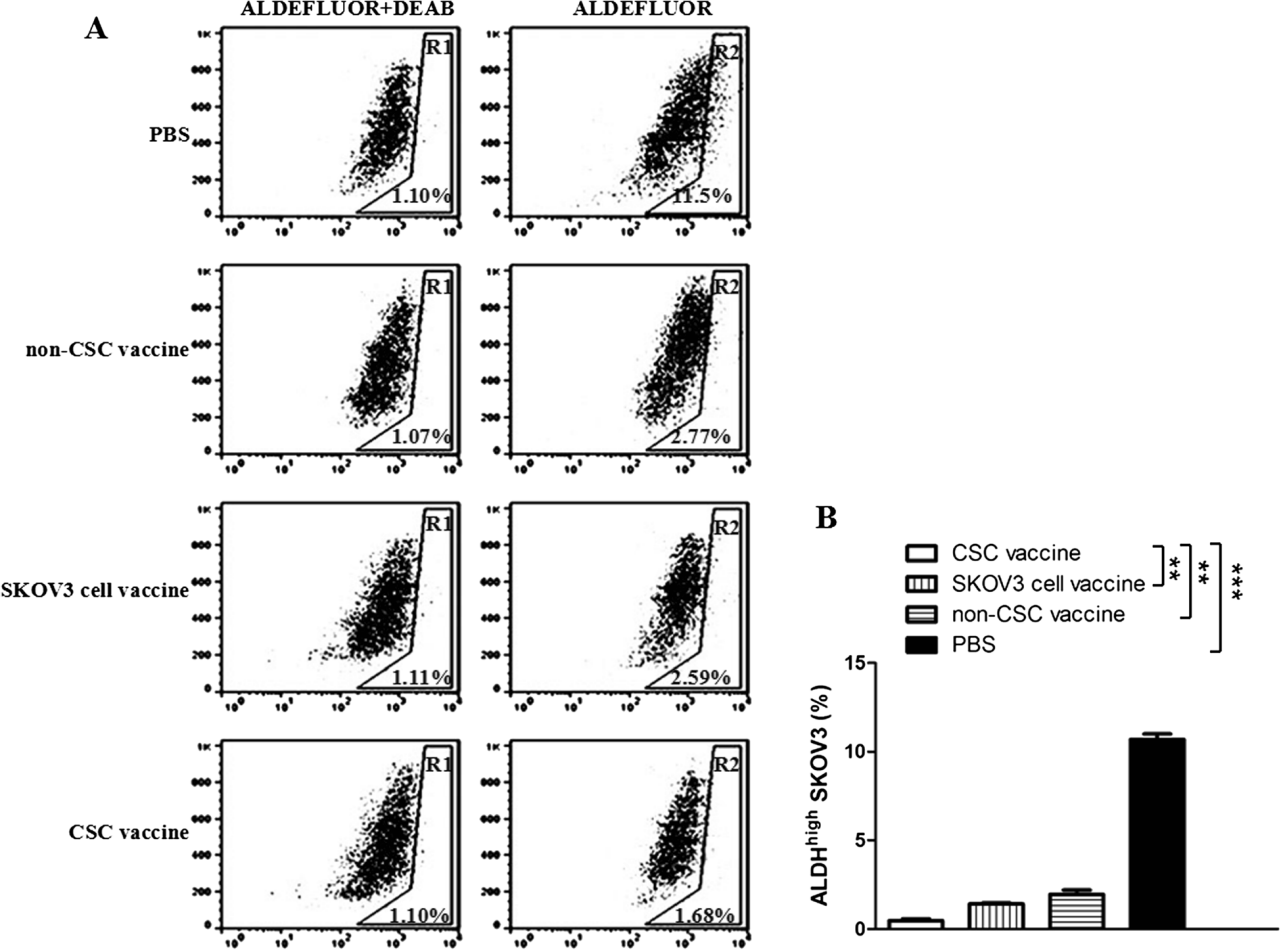
CD117^+^CD44^+^CSC vaccine decreases ALDH-positive cells in ovarian cancer tissues in mice. Representative FCM analysis of ovarian cancer tissue cells (2 × 10^5^) using the ALDEFLUOR assay. The sorting gates were established based on DEAB stained controls. DEAB were used to establish the baseline fluorescence of these cells (R1) and to define the ALDEFLUOR positive cell region (R2). **a** FCM analysis shows that the number of ALDH positive cells in CD117^+^CD44^+^CSC vaccine group was the lowest among 4 groups, whereas this number was the highest in PBS group; **b** Quantitative analysis of ALDH positive cell population in ovarian cancer tissues from the mice immunized with the different vaccines. The number of ALDEFLUOR positive cell equals to the number in R2 region minus the number in R1 region. **p <0.01 and ***p <0.003; refer to the statistically significant differences as indicated

Because the ALDEFLUOR-positive cells have stem cell characteristics, and may represent CSC population [1, 22–24], we further analyzed the ALDH activity in cells derived from a SKOV3 ovarian tumors detached from the vaccinated nude mice. In Fig. 5a, FCM analysis of the xenografted tumor cells showed that ALDEFLUOR-positive cells in CD117^+^CD44^+^CSC vaccine group was accounting for 0.58 % (1.68 % minus 1.10 %), and it was remarkably decreased in contrast to the SKOV3 cell vaccine group for 1.48 % (2.59 % minus 1.11 %, **p < 0.01) or non-CSC vaccine group for 1.7 % (2.77 % minus 1.07 %, **p < 0.01) or PBS group for 10.4 % (11.5 % minus 1.10 %, ***p < 0.003) as is shown in Fig. 5b. It is thus evident from these results that the administration of the CD117^+^CD44^+^CSC vaccine to mice led to disease of the CD44^+^ CD117^+^CSC and ALDH-positive cell populations in ovarian cancer tiuuses from immunized mice.

## Discussion

EOC still belongs to the most aggressive cancer types such as high-grade serous ovarian cancer, a devastating disease with highly recurrence. Surgery and chemotherapy with taxanes and platinum compounds are very effective in reducing tumor burden, however, relapses and drug resistance occur frequently. EOC CSCs are thought to drive the onset of tumor recurrence, distant metastasis, and drug-resistance, which is a significant clinical problem for the effective treatment of cancer [2, 3, 25–27]. Thus, targeted treatment of EOC CSC modalities is eagerly awaited.

To target CSCs for treatment of EOC, we have developed the SKOV3 CD117^+^CD44^+^CSC vaccine to test this assumption. The data from our courrent study demonstrated that the CD117^+^CD44^+^ CSC vaccine were able to induce athymic nude mice for generating immune responses against human EOC SKOV3 cell challenge in the vaccinated mice. Although the non-CD117^+^CD44^+^CSC and the SKOV3 cell vaccines showed marked efficacy against ovarian cancer as well, this efficacy was actually more efficient in the mice immunized with the CD117^+^CD44^+^CSC vaccine. The efficacy mechanisms, we guess, may involve in the elevated serum IFN-γ level, and the enhanced the cytotoxic activity of NK cells. IFN-γ was generated by NK cells, while IFN-γ again reacted on NK cells, which may enhance cell-mediated cytotoxicity by delivering perforin and granzyme B, and develop central biological role in killing ovarian cancer cells [20, 28, 29]. Differently, the malignant tumors secreted the high amounts of TGF-β, which increased circulating plasma concentration that is associated with the advanced stage of the tumors [30–32]. The dysregulation of TGF-β signaling plays a crucial role in ovarian carcinogenesis and maintaining CSC properties [33]. In this study, we found that CD117^+^CD44^+^CSC vaccine significantly suppressed the secretion of TGF-β in ovarian cancer tissues, which may be one of anti-ovarian cancer mechanisms by inhibition of ovarian carcinogenesis and regulating CSC properties.

Because the numbers of CD117^+^CD44^+^CSCs and the ALDEFLUOR-positive cell populations that have self-renew characteristics, are closely related with the sensitivity of ovarian cancer to chemotherapy and radiotherapy as well as patients survival time [34, 35], we measured the ALDEFLUOR-positive cell changes in the vaccinated mice to analyze the CSC vaccine efficient mechanisms. The results demonstrated that the CD117^+^CD44^+^CSC vaccine not only markedly decreased the CD117^+^CD44^+^CSC population, but also reduced ALDH-positive cell population in SKOV3 ovarian cancer tissues from the vaccinated nude mice compared with the mice vaccinated with other control vaccines. Consistent with the ALDEFLUOR-positive cell population, the tumors generated by this population occured earlier and grew bigger in PBS vaccinated mice than that of mice vaccinated with other vaccines. These positive consistent data allows us suppose that our developed SKOV3 CD117^+^CD44^+^CSC vaccine induced anti-ovarian cancer efficacy that is related with the diminution of CD117^+^CD44^+^CSC as well as ALDH-positive cell populations by eliciting effective immunity in the athymic nude mouse model.

At present time, there are the reports on effective immunity against ovarian cancer with xenogeneic poly antigenic cancer vaccines. These studies have demonstrated an efficacy of such vaccine with heat shock protein 70 and tumour dendritic cell fusions that targeted resistant CSC population or using fusions of dendritic cells and ovarian cancer-initiating cells that induced the cytotoxic T lymphocytes against ovarian cancer-initiating cells [36, 37]. The similar studies such as vaccination with human embryonic stem cells or mouse embryonic stem cells demonstrated that this pre-inactivated human or mouse embryonic stem cell vaccine can induce anti ovarian cancer efficacy in mouse and rat animal models, indicating that the activity of the vaccine is universal, and, more importantly, it is safe and has a potential for ovarian cancer [38]. However, to the best of our knowledge, it is first report that we used the human SKOV3 CD117^+^CD44^+^CSC vaccine to directly immunize the nude mice for evaluating vaccine efficacy against EOC CSCs. Nevertheless, we understand that more studies are fully warranted to find out the mechanisms for this vaccine before SKOV3 CD117^+^CD44^+^CSC-based vaccine is moved into clinical testing. For example, why the SKOV3 CD117^+^CD44^+^CSC vaccine efficacy is better than that of SKOV3 non-CD117^+^CD44^+^CSC and the SKOV3 cell vaccines, and what molecules elicit a powerful immune responses in this CSC-based vaccine? Thus, such mechanism requires further studies.

In summary, this is a preliminary study that is the first proof for demonstrating the SKOV3 CD117^+^CD44^+^CSC vaccine targets effectively CSCs and inhibits ovarian tumor growth in xenografted nude mice by eliciting effective immune resonses against SKOV3 CD117^+^CD44^+^ CSCs. This CSC-based vaccine may confer an effective immunity against ovarian cancer.
